# 2887. Implication of genotypes for prognosis of *Candida glabrata* bloodstream infections

**DOI:** 10.1093/ofid/ofad500.164

**Published:** 2023-11-27

**Authors:** Pao-Yu Chen, Yu-Shan Huang, Yu-Chung Chuang, Jann-Tay Wang, Wang-Huei Sheng, Yee-Chun Chen, Shan-Chwen Chang

**Affiliations:** National Taiwan University Hospital, Tapei, Taipei, Taiwan (Republic of China); National Taiwan University Hospital, Tapei, Taipei, Taiwan (Republic of China); National Taiwan University Hospital and National Taiwan University College of Medicine, Taipei, Taipei, Taiwan; National Taiwan University Hospital, Tapei, Taipei, Taiwan (Republic of China); National Taiwan University Hospital, Taipei, Taipei, Taiwan (Republic of China); National Taiwan University Hospital, Taipei, Taipei, Taiwan (Republic of China); National Taiwan University Hospital, Taipei, Taipei, Taiwan (Republic of China)

## Abstract

**Background:**

Genotyping a specific pathogen may demonstrate unique patterns of antimicrobial resistance and/or interaction between pathogen and host, which subsequently lead to miserable consequence. Herein, we conducted a retrospective single-center study to analyze the association between genotypes and clinical outcomes among *Candida glabrata* bloodstream infections (BSIs).

**Methods:**

A standard case report form was used to collect the clinical data of hospitalized adults with *C. glabrata* BSIs in 2017. Antifungal susceptibility testing was performed by using the Sensititre YeastOne SYO-10 panel, and minimum inhibitory concentrations (MICs) were interpretated according to the Clinical and Laboratory Standard Institute. Genotyping was performed by using a multilocus sequence typing scheme, and further analyzed by the unweighted pair group method with arithmetic averages (UPGMA) method.

**Results:**

Among 48 patients, clonal complex 7 (CC7, defined by UPGMA similarities >80%) was the most common CC (n=14, 29.2%). The rates of fluconazole and candin resistance were low (6.6% and 0%, respectively) without specific distributions among genotypes Charlson comorbidity index (adjusted odd ratio [aOR], 1.49; 95% CI, 1.05-3.11) was the only risk factor for CC7 *C. glabrata* BSIs. CC7 was independently associated with 28-day mortality (aOR, 5.88; 98% CI, 1.06-32.47) in addition to a APACHE II score of >18 (aOR, 5.84; 98% CI, 1.16-29.46). The Kaplan–Meier survival analysis also showed greater mortality in CC7 (**Figure**). Fluconazole resistance or candin therapy had no significant impact on mortality.

**Figure.**
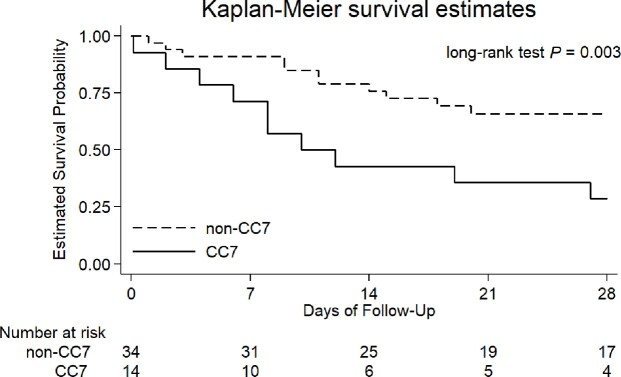
Comparison of Kaplan–Meier survival curves between cloncal complex 7 (CC7) and non-cloncal complex 7 (non-CC7) at 28 days in patients with Candida glabrata bloodstream infections

**Conclusion:**

Our data shed light on the impact of genotypes on clinical outcomes in *C. glabrata* BSIs. Further virulence characterization of CC7 is warranted.

**Disclosures:**

**All Authors**: No reported disclosures

